# Real-Time Flash Glucose Monitoring Had Better Effects on Daily Glycemic Control Compared With Retrospective Flash Glucose Monitoring in Patients With Type 2 Diabetes on Premix Insulin Therapy

**DOI:** 10.3389/fendo.2022.832102

**Published:** 2022-02-10

**Authors:** Reng-na Yan, Ting-ting Cai, Lan-lan Jiang, Ting Jing, Ling Cai, Xiao-jing Xie, Xiao-fei Su, Lan Xu, Ke He, Liang Cheng, Cheng Cheng, Bing-li Liu, Yun Hu, Jian-hua Ma

**Affiliations:** ^1^ Department of Endocrinology, Nanjing First Hospital, Nanjing Medical University, Nanjing, China; ^2^ Department of Endocrinology, Wuxi People’s Hospital Affiliated to Nanjing Medical University, Wuxi, China; ^3^ Department of Endocrinology, Wuxi Hospital of Traditional Chinese Medicine, Wuxi, China; ^4^ Department of Endocrinology, Huai’an Second People’s Hospital and the Affiliated Huai’an Hospital of Xuzhou Medical University, Huai’an, China; ^5^ Department of Endocrinology, The Affiliated Suqian First People’s Hospital of Nanjing Medical University, Suqian, China

**Keywords:** type 2 diabetes, flash glucose monitoring, premix insulin, time in target range, real-time glucose monitoring

## Abstract

**Background and Aims:**

To compare the effects of real-time and retrospective flash glucose monitoring (FGM) on daily glycemic control and lifestyle in patients with type 2 diabetes on premix insulin therapy.

**Methods and Results:**

A total of 172 patients using premix insulin, with HbA1c ≥ 7.0% (56 mmol/mol), or the time below the target (TBR) ≥ 4%, or the coefficient of variation (CV) ≥36% during the screening period, were randomly assigned to retrospective FGM (n = 89) or real-time FGM group (n = 83). Another two retrospective or real-time 14-day FGMs were performed respectively, 1 month apart. Both groups received educations and medication adjustment after each FGM. Time in range (3.9~10.0 mmol/l, TIR) increased significantly after 3 months in the real-time FGM group (6.5%) compared with the retrospective FGM group (-1.1%) (*p* = 0.014). HbA1c decreased in both groups (both *p* < 0.01). Real-time FGMs increased daily exercise time compared with the retrospective group (*p* = 0.002).

**Conclusions:**

Real-time FGM with visible blood glucose improves daily glycemic control and diabetes self-care behaviors better than retrospective FGM in patients with type 2 diabetes on premix insulin therapy.

**Clinical Trial Registration:**

https://clinicaltrials.gov/NCT04847219.

## Introduction

Effective self-management, such as self-monitoring of blood glucose (SMBG), diet, and physical activity, is foundational to achieving treatment goals for patients with diabetes (1). SMBG is a cornerstone of diabetes self-care, which provides information about current glycemic status, guiding adjustments in diet, exercise, and medication (2). SMBG is especially important for insulin-treated patients to monitor for and prevent hypoglycemia and hyperglycemia (3). However, the frequency of SMBG is commonly low in these patients due to the fear of needles and pain, inconvenience, and unconducive environment for testing (4).

The flash glucose monitoring (FGM) system is a new glucose testing device, which displays an estimate of blood glucose every 15 min and can be scanned for a glucose reading at any time with a long sensor lifetime of 14 days and no need for calibration. There are currently two types of FGM system produced by Abbott Diabetes Care, FreeStyle Libre™ and FreeStyle Libre Pro™. The main difference of these two modes of FGM is that the FreeStyle Libre Pro™ (blinded mode) can mask the glucose levels to patients and reduce the behavior change of patients during glucose monitoring; therefore, clinicians can identify and correct patterns of hyper- and hypoglycemia in patients with diabetes; FreeStyle Libre™ (unblinded mode) provides real-time glucose levels to patients and encourages patients for their diet, exercise, or medication change according to glucose levels immediately. Both of these two modes of FGM are wildly used in patients with diabetes in China.

Previous studies have demonstrated that both blinded and unblinded FGM can improve glycemic control in patients with type 2 diabetes (T2DM) compared with SMBG (5–7), and the main reason was that FGM guided the adjustment of insulin dosage or oral antidiabetic drugs in these patients. Our previous study showed that blood glucose improved during 14 days of unblinded FGM without change of antidiabetic drugs in patients with T2DM (8, 9). We hypothesized that the improvement of blood glucose contributed to the effect of unblinded FGM on self-care behavior, which was also indicated by White et al. (10). However, there was no strong evidence to support our hypothesis yet as we are aware of.

Premix insulins have been widely used worldwide. The MOSAIc study of 18 countries showed that about 30% of people with T2DM taking insulin were using premix insulin globally, and the percentage was 67% in China (11). However, several real-world studies have shown that glycemic control remains unsatisfactory 6–12 months after initiating or switching therapy with premix insulin (12–14). The reasons of poor glycemic control in patients on premix insulin include fear of weight gain and hypoglycemia and the need for frequent self-monitoring of blood glucose (12). FGM may be a good solution to these problems.

Therefore, we performed this randomized controlled study to investigate the effects of real-time FGM (unblinded FGM) on daily glycemic control and the changes of diet and exercise in patients with type 2 diabetes who were on premixed insulin therapy, and we used retrospective FGM (blinded FGM) as control to exclude the effects of drug adjustment from doctors.

## Research Design and Methods

### Participants

This trial was conducted at 5 diabetes centers in Jiangsu, China, from October 2019 to April 2021.

Patients with type 2 diabetes, who were treated with premix insulin, two or three injections a day, single drug or combination of oral hypoglycemic drugs, and whose treatment regimen was stable for more than 2 months, were considered eligible to be enrolled in the study. Exclusion criteria were the following: (1) patients treated with GLP-1 agonist or any other drugs that may affect appetite in the last 3 months; (2) allergic to insulin; (3) impaired liver and renal function (ALT 2.5 times higher than the upper limit of normal value; serum creatinine was 1.3 times higher than the upper limit of normal); (4) a history of drug abuse and alcohol dependence; (5) used systemic glucocorticoid therapy in the recent 3 months; (6) patients with infection or stress within 4 weeks; (7) patients who cannot tolerate FGM; (8) pregnant or preparing to become pregnant; and (9) considered unsuitable to participate by the investigator.

### Study Design

**Figure 1:**
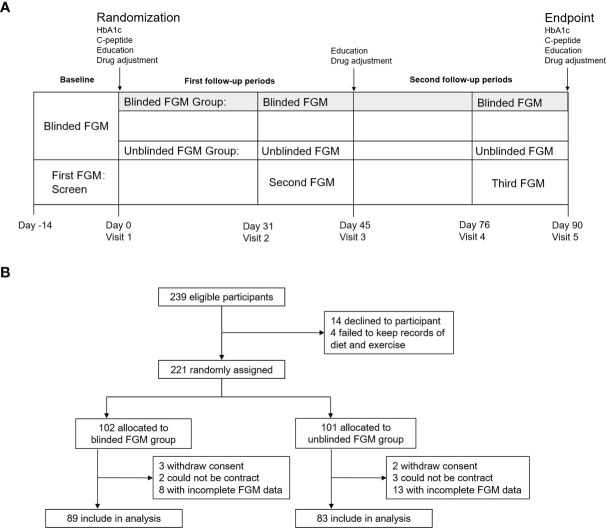
Study design **(A)** and trial profile **(B)**.

This is a prospective, randomized controlled trial. At baseline, all participants were screened by a blinded FGM for 14 days and a glycosylated hemoglobin (HbA1c). Patients were enrolled when their HbA1c ≥ 7.0% (56 mmol/mol), or the FGM showed that the percentage time spent in hypoglycemia ≤ 3.9 mmol/l (time below the target range, TBR) ≥ 4% or the coefficient of variation (CV) ≥ 36% (15). Then the patients were randomized into blinded FGM and unblinded FGM groups in a 1:1 ratio. All participants were educated by a diabetes specialist nurse. The content of education included the insulin injection technique and self-management of diet and exercise. Diabetes clinicians adjusted the antidiabetic drugs according to the results of FGM and the guideline of care for type 2 diabetes in China (16). Then the participants entered into two successive 45-day follow-up periods (**Figure 1A**) . Both of the groups performed an FGM during the last 14 days of each follow-up period, and educations and drug adjustment were taken immediately after each FGM. The educators and clinicians were not told and should not ask the patients about the type of FGM. Moreover, the results of both FGM modes were reported in the same format.

Ethics Committee approval was granted prior to the study. All procedures were in accordance with the Helsinki Declaration. All patients provided written informed consent forms to participate in the study. This trial is registered with ClinicalTrials.gov (NCT04847219).

### Flash Glucose Monitoring

FreeStyle Libre™ and FreeStyle Libre Pro™ (Abbott Diabetes Care, Maidenhead, UK) were used in the unblinded and blinded groups, respectively. The sensor was worn on the back of the left upper arm for 14 days to record the subcutaneous interstitial glucose concentration at 15-min intervals. For the blinded FGM group, the results of blood glucose in blinded FGM were masked, and patients could take SMBG at any time. For the unblinded FGM group, patients could scan the sensor to read the glucose levels at any time, but they have to scan the sensor every 8 h at least. Patients in both groups were required to keep track of their food intake and exercise while wearing the FGM sensor and could alter their diet and exercise according to the glucose levels. We dispensed a uniform study log for patients to record their diet and exercise for all days during FGMs, including the type (write the names of food) and weight of each food and when it was eaten, and the type of exercise, and the time when the exercise began and ended. However, patients could not change their therapy with glucose-lowering agents during FGM. The dosage of glucose-lowering agents could be adjusted by clinicians according to the results of the FGM when the FGM sensors were removed.

### Clinical and Laboratory Assessment

The height, duration of disease, concomitant diseases, and change of weight and medications of all patients were recorded. Blood samples of all patients were collected after overnight fasting (>10 h). Fasting C-peptide and HbA1c were measured immediately after the first and third FGMs. All tests were performed in the Nanjing Clinical Nuclear Medicine Center (ISO/IEC15189/17020).

### Outcomes

The primary outcome of this study was the change in percentage time in the target range (glucose 3.9~10.0 mmol/l, TIR) between the first (baseline) and third (endpoint) FGMs. Secondary outcomes included TBR, percentage time spent in hyperglycemia > 10.0 mmol/l (time above target range, TAR), 24 h mean blood glucose (MBG), standard deviation of blood glucose (SDBG), CV, hourly mean blood glucose (the average of 14 days during FGM), HbA1c, C-peptide, and daily exercise time, energy intake, number of meals, and insulin dose per day during FGM.

### Statistical Analysis

All statistical analyses were performed using SPSS version 22.0 software (IBM Corp., Foster City, CA, USA). All variables were tested for normal distribution. Data are presented as mean (95% CI) or percentage. Differences between the two groups were examined using Student’s unpaired *t-*test (insulin dose) or the Mann–Whitney *U*-test (age, diabetic duration, BMI, exercise time, carbohydrate, calories, and daily meal frequency at baseline). The parameters (TIR, MBG, CV, and SDBG) assessed by three FGMs, and HbA1c, C-peptide, insulin, and metformin dose, and lifestyles at baseline and endpoint were analyzed by a mixed-model ANOVA with time as the within-subject factor and groups as the between-subject factor. The categorical data were examined with the chi-square test. All comparisons were 2-sided at a 5% significance level. A p value < 0.05 was considered statistically significant.

This study is registered with ClinicalTrials.gov, number NCT04847219.

## Results

There were 239 eligible patients and 221 patients finished the screening phase. Among these patients, 74 (33.5%) patients had HbA1c<7%, 141 (63.8%) patients had CV<36%, and 131 (59.3%) patients had TBR<4%. Therefore, only 18 (8.1%) patients achieved the composite goal of glycemic control including HbA1c, CV, and TBR, and the other 203 patients were randomized into two groups. There were 8 patients in the blinded FGM group, and 13 patients in the unblinded FGM group failed to complete the two FGMs after randomization because of sensors falling off or data missing. Finally, there were 89 patients in the blinded FGM group and 83 patients in the unblinded FGM group included for analysis (**Figure 1B**). Participant characteristics at baseline were similar between the study groups except insulin dose and the percentage of acarbose use (**Table 1**).

**Table 1:** Baseline characteristics of participants.

	Blinded FGM Group	Unblinded FGM Group	*p* value*^a^*
Age (year)	63.8 (61.7,65.9)	61.3 (59.3,63.3)	0.083
Gender (male)	54 (60.7%)	56 (67.5%)	0.427
Diabetic duration (month)	162.9 (144.9,181.0)	164 (145.3,182.7)	0.711
BMI (kg/m^2^)	25.2 (24.6,25.9)	24.7 (24,25.4)	0.355
*Glucose-lowering drugs*			
Insulin dose (IU/day)	39.3 (36.8,41.9)	35.4 (32.8,38.0)	0.034
Metformin (%)	37 (41.6%)	37 (44.6%)	0.759
Acarbose (%)	33 (37.1%)	19 (22.9%)	0.048
Insulin secretagogues (%)	6 (6.7%)	4 (4.8%)	0.748
DPP-4 inhibitors (%)	4 (4.5%)	8 (9.6%)	0.237
TZDs (%)	2 (2.2%)	0 (0%)	0.498
*Diabetic complications*			
Diabetic kidney disease (%)	16 (18.0%)	9 (10.8%)	0.201
Neuropathy (%)	13 (14.6%)	11 (13.3%)	0.829
Retinopathy (%)	14 (15.7%)	18 (21.7%)	0.334
Coronary heart disease (%)	17 (19.1%)	17 (20.5%)	0.850
Cerebral infarction (%)	19 (21.3%)	16 (19.3%)	0.850
*Lifestyle*			
Exercise time (min/day)	66.3 (55.2,77.4)	62.4 (51.6,73.2)	0.490
Calories/weight daily (kcal/kg)	25.8 (23.4,28.1)	27.9 (25.5,30.3)	0.174
Mean calories per meal (kcal)	492.1 (444.3,539.8)	535.2 (490.6,579.9)	0.191
Carbohydrate (g)/day)	270.2 (251.6,288.9)	275.5 (259.1,291.2)	0.726
Meal frequency daily (number)	3.6 (3.4,3.7)	3.5 (3.4,3.7)	0.639

Data are mean (95% CI) or number (percentage).

^a^Difference between two groups with the Mann–Whitney U-test or chi-square test.

FGM, flash glucose monitoring; BMI, body mass index; DPP-4, dipeptidyl peptidase 4; TZDs, thiazolidinediones.

### The Changes of Daily Glycemic Control

**Figure 2:**
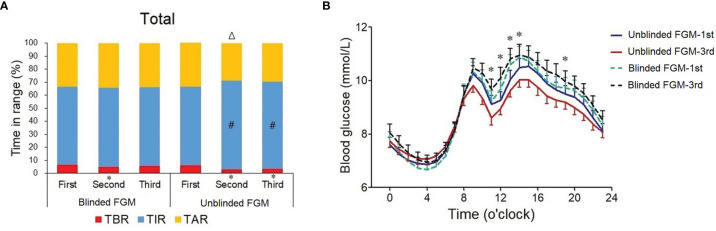
Changes in daily glycemic control during three flash glucose monitorings in professional and unblinded FGM groups. **(A)** Percentage time in the target range of 3.9–10.0 mmol/l (TIR) during the first (baseline, blinded FGM in both groups), second (days 31–45), and third (days 76–90) FGM in the blinded FGM group (n = 89) and unblinded FGM group (n = 83), blue bar; time below the target range (TBR), red bar; time above the target range (TAR), yellow bar. Data are percentage; #, vs. first FGM, *p value <*0.05. **(B)** Hourly mean blood glucose during the first and third FGMs. Blue solid line, first FGM in the unblinded FGM group; red solid line, third FGM in the unblinded FGM group; green dotted line, first FGM in the blinded FGM group; black dotted line, third FGM in the blinded FGM group. *p < 0.05 between two groups.

There were no differences of daily glycemic control (p all >0.05), HbA1c (p = 0.990), and C-peptide (p =0.420) between the blinded and unblinded FGM groups at baseline during the first blinded FGM (**Table 2**). A mixed-model ANOVA showed that TIR increased significantly in the second and third FGMs in the unblinded FGM group (p < 0.001) but did not change in the blinded FGM group (p = 0.709). Therefore, a difference of TIR change appeared between the two groups (estimated treatment difference -7.7 (-13.9,1.4) %, p = 0.014), and the difference remained significant after adjusting for insulin and acarbose dose at baseline (p = 0.031, **Table 2**). Both unblinded FGM showed a higher TIR than baseline (both p < 0.05, **Figure 2A**). TBR, CV, SDBG, and HbA1c were significantly decreased (p all <0.05), and SDBG was lower in the unblinded group than in the blinded group (p = 0.029, **Table 2**).

**Table 2:** Changes of daily glycemic control in blinded and unblinded FGMs.

			Blinded FGM	Unblinded FGM	Estimated Treatment Difference	p value (Time)	p value (Group)	p value (Time × Group)	Adjusted *p* value *^a^* (Time × Group)
TIR (%)	First		60.6 (56.1,65.0)	60.6 (56.4,64.8)	-7.7 (-13.9,1.4)	0.010	0.056	0.014	0.031
	Second		61.0 (56.6,65.4)	68.2 (64.2,72.3)					
	Third		59.4 (54.3,64.6)	67.1 (63.1,71.1)					
Endpoint—baseline			-1.1(-5.9, 3.6)	6.5 (2.4, 10.6)					
	p value		0.709	<0.001					
TBR (%)	First		6.5 (4.8,8.2)	5.9 (3.8,7.9)	1.4 (-1.0,3.7)	0.007	0.132	0.222	0.320
	Second		4.7 (3.1,6.3)	3.1 (2.3,3.9)					
	Third		5.5 (3.8,7.1)	3.5 (2.5,4.4)					
Endpoint—baseline			-1.0(-2.7, 0.6)	-2.4 (-4.2, -0.6)					
TAR (%)	First		32.9 (27.7,38.1)	33.6 (28.7,38.5)	6.3 (-0.5,13.1)	0.400	0.215	0.072	0.110
	Second		34.3 (29.5,39.1)	28.7 (24.4,32.9)					
	Third		35.1 (29.3,40.9)	29.5 (25.1,33.8)					
Endpoint—baseline			2.2(-3.0, 7.4)	-4.1 (-8.6,0.3)					
MBG (mmol/L)	First		8.9 (8.3,9.5)	8.8 (8.3,9.3)	0.4 (-0.2,1.1)	0.915	0.215	0.232	0.294
	Second		9.0 (8.5,9.5)	8.6 (8.2,8.9)					
	Third		9.2 (8.5,9.8)	8.6 (8.2,9.0)					
Endpoint—baseline			0.2(-0.3,0.8)	-0.2 (-0.6,0.2)					
CV (%)	First		35.3 (33.9,36.7)	33.9 (32.4,35.4)	0.6 (-1.2,2.4)	0.001	0.057	0.346	0.464
	Second		34.4 (32.8,35.9)	32.3 (30.9,33.7)					
	Third		33.8 (32.3,35.4)	31.8 (30.4,33.2)					
Endpoint—baseline			-1.5(-2.8, -0.2)	-2.1 (-3.4, -0.8)					
SDBG (mmol/L)	First		3.1 (2.9,3.2)	2.9 (2.8,3.1)	0.2 (-0.05,0.4)	0.007	0.029	0.105	0.185
	Second		3.0 (2.9,3.2)	2.8 (2.6,2.9)					
	Third		3.0 (2.8,3.2)	2.7 (2.6,2.9)					
Endpoint—baseline			-0.1(-0.2,0.1)	-0.2 (-0.4,-0.1)					
HbA1c (%)		Baseline	7.5 (7.3,7.8)	7.6 (7.3,7.8)	0.1 (-0.2,0.4)	<0.001	0.851	0.563	0.752
		Endpoint	7.3 (7.0,7.5)	7.2 (7.0,7.4)					
Endpoint—baseline			-0.3(-0.5,-0.1)	-0.4 (-0.5,-0.2)					
C-peptide (ng/mL)		Baseline	1.5 (1.2,1.7)	1.4 (1.2,1.6)	0.01 (-0.3,0.3)	0.444	0.561	0.213	0.817
		Endpoint	1.5 (1.1,1.9)	1.4 (1.2,1.6)					
Endpoint—baseline			0.06(-0.2,0.3)	0.06 (-0.1,0.2)					

Data are mean (95% CI).

a Adjusted for baseline insulin and acarbose dose in mixed-model ANOVA analysis.

FGM, flash glucose monitoring; TIR, time in target range; TBR, time below target range; TAR, time above target range; MBG, mean blood glucose; CV, coefficient of variation; SDBG, standard deviation of blood glucose.

The hourly mean blood glucose over 14-day FGM periods showed that the blood glucose levels after meals were lower in the unblinded FGM group during the third FGM, especially after lunch (11:00~14:00) and supper (19:00) (p all <0.05, **Figure 2B**), which were similar between the two groups during the first FGM (p all >0.05). There was no difference of nocturnal blood glucose between unblinded and blinded FGMs (p all >0.05, **Figure 2B**). However, the changes of hourly mean blood glucose from the first FGM to the third FGM in each group were not statistically significantly according to the t-test (p all >0.05).

### The Changes of Medications and Lifestyle

To explore the factors which may influence the daily glycemic control in different groups, changes (endpoint minus baseline) of medications and lifestyles were compared between blinded and unblinded FGM groups. As a result, the changes of insulin and metformin dose and the proportions of oral antidiabetic agents used at endpoint were all similar in the two groups (p all >0.05, **Table 3** and **Supplementary Table 1**).

**Table 3:** Changes of medications, and lifestyles from baseline to endpoint.

	Blinded FGM (Endpoint-Baseline)	Unblinded FGM (Endpoint-Baseline)	p value (time)	p value (Group)	p value (Time × Group)
Insulin dose (IU/day)	-1.0 (-2.3,0.3)	-2.3 (-3.7,-0.9)	0.001	0.065	0.152
Metformin dose (g/day)	-0.1 (-0.4,0.1)	0.1 (-0.02,0.2)	0.160	0.370	0.109
Exercise time (min/day)	-10.1 (-19.5,-0.6)	8.0 (1.1,14.8)	0.716	0.312	0.002
Calories/weight daily (kcal/kg)	1.7 (-0.3,3.8)	-0.04 (-1.9,1.8)	0.225	0.393	0.338
Mean calories per meal (kcal)	47.5 (5.7,89.2)	19.7 (-21.6,61.0)	0.024	0.339	0.347
Carbohydrate (g)/day)	-5.3 (-22.9,12.3)	-7.0 (-19.9,5.9)	0.263	0.766	0.880
Daily meal frequency (number)	-0.13 (-0.26,0.003)	-0.08 (-0.21,0.04)	0.022	0.641	0.607

Data are mean (95% CI); data were analyzed by a mixed-model ANOVA.

DPP-4, dipeptidyl peptidase 4; TZDs, thiazolidinediones.

As shown in **Table 3**, the daily exercise time increased to 8.0 min every day in the unblinded FGM group [62.4 (51.6, 73.2) vs. 79.0 (63.9, 94.1) min], which decreased to 10.1 min in the blinded group (66.3 (55.2, 77.4) vs. 64.0 (51.4, 76.6) min), p = 0.002. Mean calories per meal increased and daily meal frequency decreased at the endpoint compared with baseline (both p <0.05). However, the changes in calorie intake and daily meal frequency were not significantly different between the two groups (p all >0.05, **Table 3**).

### Different Changes of Daily Glycemic Control, Medications, and Lifestyle in Patients With Different Problems of Glucose Control

Since we included not only patients with hyperglycemia (HbA1c ≥7%) but also patients with hypoglycemia (TBR ≥4%) or high glycemic variability (CV ≥36%), we analyzed the changes of daily glycemic control in patients with each of these problems separately. There were no significant differences of time × group interaction in the mixed-model ANOVA analysis of daily glycemic control (p all >0.05, **Supplementary Figure 1**). In patients with TBR ≥4%, TIR in the blinded group was lower than in the unblinded group (p = 0.048). HbA1c decreased compared with baseline only in patients with HbA1c ≥7% (p < 0.001, **Supplementary Table 2**).

In patients with high TBR or CV, insulin dose decreased significantly at the endpoint compared with baseline (p < 0.001 and p = 0.006, respectively), and the reduction of insulin dose in the unblinded FGM group was more than in the blinded FGM group (p = 0.007 and 0.022, respectively). Moreover, patients had higher elevation of calorie intake/weight and mean calories per meal (p = 0.045 and 0.047, respectively) and higher reduction of exercise time (p = 0.007) than in the unblinded FGM group in patients with TBR ≥ 4% (**Supplementary Table 2**).

## Discussion

Although both FGM modes improved HbA1c significantly in patients using premix insulin in the present study, the TIR and parameters that reflect glycemic variability improved better in the unblinded FGM group than in the blinded FGM group. The essence of this result is that on the basis of clinicians’ adjustment of diabetic therapy according to retrospective FGM data once a month, patients can improve their blood glucose better, modulating their diet and exercise according to visible FGM data more effectively compared with regular SMBG. By using the retrospective FGM as a control, the present study was able to compare TIR between the two groups and partially eliminated the interference of physician-led drug adjustment, both of which have not been discussed in previous studies comparing FGM with SMBG (17–19).

The interventions in the blinded FGM group were almost the current pattern of outpatient follow-up for patients with diabetes in China. Patients come to the hospital once a month, and doctors give advices about diet, exercise, and medications according to their SMBG records during the last month. The CCMR-3B study in China showed that 47.7% outpatients with T2DM achieved the target goals for the control of blood glucose (HbA1c <7%) (20), and the proportion in the present study was even lower in patients using premix insulin. Moreover, only 8.1% patients achieved the composite goal of glucose control with additional combination of hypoglycemia and CV in the screening period of this study.

Before the endpoint, patients in both groups received two times of diabetic education and drug adjustment. HbA1c was reduced in both groups; however, TIR in the last blinded FGM did not improve significantly. One reason may be that the effect of education and drug adjustment cannot last for long due to the poor adherence of these patients. The fall after rise of the efficacy of blinded FGM also existed in previous studies (5, 6). On the other hand, nearly half of the patients in this study had hypoglycemic or high glycemic variability. Asymptomatic hypoglycemia was shown to these patients by the first two blinded FGMs. Therefore, the less exercise time compared with the unblinded FGM group during the last FGM may be associated with their prevention of hypoglycemia. As a result, the blinded FGM group had lower TIR than the unblinded group in the TBR ≥4% subgroup. Compared with the blinded FGM group, patients during unblinded FGM had better exercise adherence and flexible mealtimes. A previous study showed that hypoglycemia during aerobic exercise was positively correlated with pre-exercise blood glucose levels (21). ADA/ACSM also recommended that in patients treated with insulin, carbohydrate should be ingested before any exercise when the pre-exercise glucose level <5.5 mmol/l (22). Patients could obtain their blood glucose levels before and after exercise easily by scanning during unblinded FGM. Therefore, we speculate that the fear of hypoglycemia may largely decrease and the effectiveness of exercise on glycemic control was also shown by unblinded FGM. On the other hand, patients using unblinded FGMs may prevent hypoglycemia by eating when they noticed a rapid drop in blood glucose, while patients in the blinded group tried to prevent hypoglycemia by eating more at each meal in the present study. As a result, the unblinded FGM group showed better TIR compared with the blinded FGM group.

Our previous study showed that the optimal frequency of scanning time required to maintain euglycemia in patients with T2DM was 11.7 times/day during unblinded FGM (8). However, according to the standards of medical care for type 2 diabetes in China 2020, the frequency of SMBG in patients using premix insulin is twice a day (fasting and before dinner), and most of the patients did not perform SMBG every day in the present study in the blinded FGM group because of glucose test strips and the fear of pain.

Ahn et al. also suggested that unblinded continuous glucose monitoring (CGM) should replace blinded CGM in the clinical management of diabetes (23). However, only one randomized controlled crossover study (24) compared the effects of blinded and unblinded CGM directly as we are aware of. In this previous study, HbA1c decreased more, less time was spent in hypoglycemia, and insulin pump was used more frequently when real-time data were available to the subjects compared with those during blinded CGM in patients with type 1 diabetes (T1DM) using insulin pump therapy. Our present study showed similar results in FGMs and extends the applicability to patients with type 2 diabetes using premix insulin with more details on the changes of diet and exercise.

Although unblinded FGM has better effects on daily glycemic control, there are still some shortcomings of unblinded FGM. Patients using an unblinded FGM must scan the sensor at least every 8 h to avoid data interruptions. As a result, the unblinded group had more data missing than the blinded FGM group (not statistically significant, **Figure 1B**). Moreover, unblinded FGM does not have alarms for hypo- and hyperglycemia. It has been demonstrated that real-time CGM with alarm was superior to FGM in reducing hypoglycemia and improving TIR in adults with T1DM with normal hypoglycemia awareness (25). However, no need for calibration remains a superiority of unblinded FGM for patients compared with real-time CGM.

Our study has several potential limitations. Although both blinded and unblinded FGMs had similar accuracy with CGM and SMBG in previous studies (26–28), head-to-head comparison of the accuracy between the two modes of FGM has not been reported yet. Therefore, we cannot exclude the uncertain influence of different accuracies in the two modes of FGMs completely, which needs to be further studied. Moreover, we used self-reported dietary and exercise data, which are normally associated with underreporting and social desirability bias (29, 30).

In conclusion, this randomized controlled trial indicates that real-time FGM with visible blood glucose can improve daily glycemic control and diabetes self-care behaviors better than retrospective FGM. Our study provides strong evidence for the use of real-time FGM/CGM instead of blinded FGM/CGM in clinical practice. In addition to clinicians’ guidance of antidiabetic medications and educations for diet and exercise during outpatient sessions, patients’ self-care based on their real-time blood glucose monitoring at home may play a more important role in blood glucose control than what we have realized.

## Data Availability Statement

The original contributions presented in the study are included in the article/**Supplementary Material**. Further inquiries can be directed to the corresponding authors.

## Ethics Statement

The studies involving human participants were reviewed and approved by the Ethics Committee of Nanjing First Hospital. The patients/participants provided their written informed consent to participate in this study.

## Author Contributions

JHM and YH are responsible for the conception and design of the study. YH carried out statistical analysis. R-nY, T-tC, TJ, LC, L-jJ, X-jX, X-fS, LX, KH, LC, and CC researched data. B-lL approved the final version of the manuscript. J-hM and YH contributed to obtain funding. J-hM is the guarantor of this work and, as such, had full access to all the data in the study and take responsibility for the integrity of the data and the accuracy of the data analysis. All authors contributed to the article and approved the submitted version.

## Funding

This study was supported by the National Natural Science Foundation of China (No. 81870563) and the fellowship of China postdoctoral Science Foundation (No. 2020M671535).

## Conflict of Interest

The authors declare that the research was conducted in the absence of any commercial or financial relationships that could be construed as a potential conflict of interest.

## Publisher’s Note

All claims expressed in this article are solely those of the authors and do not necessarily represent those of their affiliated organizations, or those of the publisher, the editors and the reviewers. Any product that may be evaluated in this article, or claim that may be made by its manufacturer, is not guaranteed or endorsed by the publisher.
