# Supplementary material for: Real-Time Flash Glucose Monitoring Had Better Effects on Daily Glycemic Control Compared With Retrospective Flash Glucose Monitoring in Patients With Type 2 Diabetes on Premix Insulin Therapy
Source: Front Endocrinol (Lausanne). 2022 Feb 10;13:832102. doi: 10.3389/fendo.2022.832102 (PMC8867069; doi:10.3389/fendo.2022.832102)
Supplement: Supplementary file 1 [file DataSheet_1.pdf]

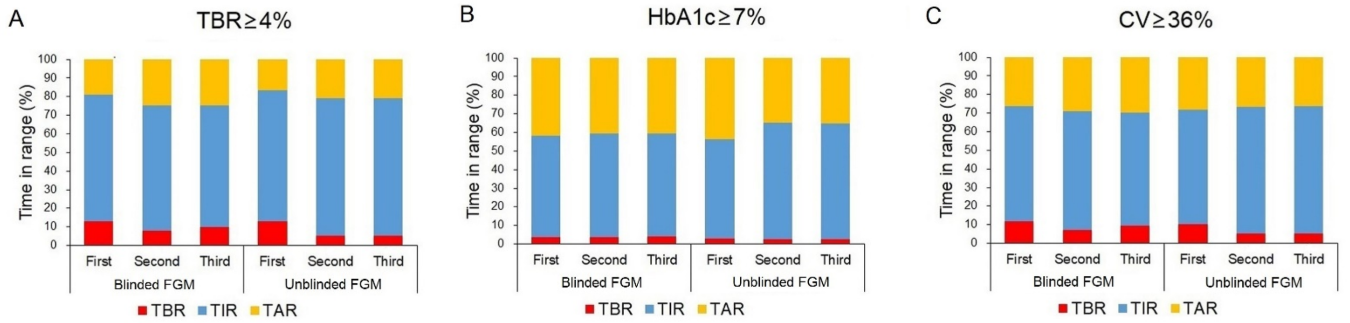

Supplementary Figure 1. Changes of daily glycemic control in different subgroup. TIR, TBR, and TAR in patients with (A)TBR ≥4%, (B)HbA1c ≥7%, and (C)CV≥36% were also showed. Data are percentage.

Supplementary table 1. The proportions of hypoglycemic medications used at the endpoint.

	Blinded FGM	Unblinded FGM	***P*** value
Metformin (%)	40(44.9%)	37(44.6%)	0.759
Acarbose (%)	25(28.1%)	22(26.5%)	0.865
Insulin secretagogues (%)	6(6.7%)	4(4.8%)	0.748
DPP-4 inhibitors (%)	18(20.2%)*	22(26.5%)*	0.369
TZDs (%)	2(2.2%)	0(0%)	0.498

Data are number (percentage); data were analyzed by chi-square tests; *, vs. baseline, $p<0.05$; DPP-4, dipeptidyl peptidase 4; TZDs, thiazolidinediones.

Supplementary table 2. Changes (endpoint minus baseline) of medications and lifestyles in patients with different problems of glucose control.

	TBR ≥4%		HbA1c ≥7%		CV ≥36%	
	Blinded	Unblinded	Blinded	Unblinded	Blinded	Unblinded
Number	40	33	60	53	31	29
HbA1c (%)	0(-0.3,0.2)	-0.2(-0.5,0.1)	-0.5(-0.8,-0.2)	-0.7(-0.9,-0.4) †	0(-0.3,0.4)	-0.4(-0.7,0)
Exercise time (min/day)	-14.8(-29.7,0.1)	7.7(-0.4,15.9)*	-6.2(-17.1,4.7)	9(-1.4,19.5)	-14(-32.5,4.5)	5.9(-4.5,16.3)
Calories/weight daily (kcal/kg)	4.1(1.4,6.8)	0(-3.2,3.2)* †	1.2(-1.5,3.9)	-1(-3.4,1.5)	1.7(-1.9,5.4)	-0.7(-3.9,2.4)
Mean calories per meal (kcal)	123.6(64.9,182.4)	33.5(-36.8,103.9)* †	4.0(-47.6,55.7)	-8.3(-60.6,44.0)	78.5(-5.3,162.2)	11.6(-64.1,87.3)
Carbohydrate (g)/day)	22(-3.7,47.8)	1.1(-15.6,17.9)	-14.3(-39,10.5)	-13.4(-31.5,4.6)	9.2(-20.9,39.3)	-3.3(-23.9,17.3)
Daily meal frequency (number)	-0.3(-0.5,-0.1)	-0.1(-0.3,0.2)	-0.1(-0.3,0.1)	-0.1(-0.3,0.1)	-0.3(-0.5,0)	-0.1(-0.3,0.1)
Insulin dose (IU/day)	-1.2(-2.9,0.5)	-5.6(-8.5,-2.6)* †	-0.7(-2.5,1.1)	-1(-2.4,0.4)	-0.4(-1.9,1.1)	-4.4(-7.6,-1.2)* †
Metformin dose (g/day)	0.1(0,0.2)	0(-0.1,0.1)	0.1(0,0.2)	0(-0.1,0.1)	0.1(0,0.2)	0(-0.1,0.1)
Metformin (n)	0	0	2	0	0	1
Acarbose (n)	5	0	-4	3	-5	3
DPP-4 inhibitors (n)	0	2	13	13	3	3

*, for time×group in the mixed model ANOVA, $P < 0.05$; †, for time in the mixed model ANOVA, $P < 0.05$
